# Water-Window X-Ray Pulses from a Laser-Plasma Driven Undulator

**DOI:** 10.1038/s41598-020-62401-4

**Published:** 2020-03-27

**Authors:** A. R. Maier, N. Kajumba, A. Guggenmos, C. Werle, J. Wenz, N. Delbos, B. Zeitler, I. Dornmair, J. Schmidt, E. M. Gullikson, F. Krausz, U. Schramm, U. Kleineberg, S. Karsch, F. Grüner

**Affiliations:** 1 Ludwig-Maximilians-Universität, Department Physik, Am Coulombwall 1, 85748 Garching, Germany; 2 Max-Planck-Institut für Quantenoptik, Hans-Kopfermann-Str. 1, 85748 Garching, Germany; 3 Center for Free-Electron Laser Science and Department of Physics Universität Hamburg, Luruper Chaussee 149, 22761 Hamburg, Germany; 4 Center for X-Ray Optics, Lawrence Berkeley National Lab, 1 Cyclotron Road, Berkeley, CA 94720 USA; 5 Helmholtz-Zentrum Dresden - Rossendorf, Institute of Radiation Physics, Bautzner Landstrasse 400, 01328 Dresden, Germany

**Keywords:** Plasma-based accelerators, Optical physics

## Abstract

Femtosecond (fs) x-ray pulses are a key tool to study the structure and dynamics of matter on its natural length and time scale. To complement radio-frequency accelerator-based large-scale facilities, novel laser-based mechanisms hold promise for compact laboratory-scale x-ray sources. Laser-plasma driven undulator radiation in particular offers high peak-brightness, optically synchronized few-fs pulses reaching into the few-nanometer (nm) regime. To date, however, few experiments have successfully demonstrated plasma-driven undulator radiation. Those that have, typically operated at single and comparably long wavelengths. Here we demonstrate plasma-driven undulator radiation with octave-spanning tuneability at discrete wavelengths reaching from 13 nm to 4 nm. Studying spontaneous undulator radiation is an important step towards a plasma-driven free-electron laser. Our specific setup creates a photon pulse, which closely resembles the plasma electron bunch length and charge profile and thus might enable novel methods to characterize the longitudinal electron phase space.

## Introduction

Since they match the intrinsic length and time scales of matter, femtosecond (fs) x-ray pulses are ubiquitously applied as an important tool in many scientific disciplines for solving previously unknown structures^1–3^ and accessing atomic and molecular dynamics^4,5^. Today, the required high-brightness x-ray beams are provided to the user community almost exclusively by large-scale synchrotron^6,7^ and free-electron laser facilities^8–11^, which represent mature and highly developed technologies, delivering well-characterized photon beams with exceptional availability and reproducibility. Due to their size and cost, however, access to these important resources is very limited.

Laser-driven sources of high brightness particle^12–16^ and x-ray beams^17^ are promising to complement established facilities by offering easy access to compact, laboratory-scale femtosecond x-rays that are intrinsically synchronized to the optical driver and thus enable sub-fs temporal resolution in pump-probe experiments. These prospects have motivated a large variety of concepts for laser-driven x-ray sources, including high-harmonic generation^18,19^ or plasma lasers^20^, as well as novel acceleration schemes, like THz acceleration^21–23^, dielectric laser acceleration^24,25^, or laser-plasma acceleration^12,13^, which can generate x-rays from laser-driven relativistic electron beams^26–33^.

Laser-plasma acceleration in particular is a promising laboratory-scale technique to generate ultra-relativistic electron beams. Here, the interaction of a terawatt-class laser with an under-dense plasma creates a density modulation, i.e., plasma wave, trailing the laser, which traps and accelerates electrons from the plasma background. The field gradients supported by the plasma wave are orders of magnitude stronger than in a conventional radio-frequency (RF) driven cavity and as a result, the acceleration distance required for typical GeV-level electron beams can be as short as a few centimetres^34–37^. In this process, the plasma wavelength, which is typically on the order of 10 microns, determines the characteristic time and length scale. It limits the length of the generated electron bunch to a few femtoseconds^38–40^, supports electron beam currents of several kA^40,41^, and provides the optical synchronisation between driver laser and electrons. Those characteristics make plasma-generated electron beams appealing for driving a next-generation compact undulator or free-electron laser^29–31^ sources.

Creating stable and reproducible high-quality electron beams from a plasma is, however, conceptually and experimentally very challenging, as the technology is still less developed compared to conventional accelerators. While the small characteristic scale of the plasma wave supports extreme temporal resolution, it also makes it very difficult to diagnose and precisely control the laser-plasma interaction. As a consequence, plasma-driven electron beams available today provide inferior beam quality compared to RF-based accelerators, especially in terms of electron beam energy spread, and typically feature a jitter in central energy of a few-percent. Since an undulator effectively acts as a brightness converter, this electron beam quality is imprinted on the generated photon pulses.

To date, only few experiments^42–47^ have experimentally shown undulator radiation from a laser-plasma accelerated electron beam. Plasma-driven undulator radiation was generated first in the visible^42^ at 740 nm with 7.5% FWHM bandwidth, using electron beams of 65 MeV energy and achieving a beam brightness of 6.5 × 10^16^ photons/s/mrad^2^/mm^2^/0.1% bandwidth. Soft x-ray undulator radiation at 17 nm central wavelength and 22% FWHM relative bandwidth was demonstrated^43^ using 200 MeV electrons, achieving a beam brightness of 1.7 × 10^17^ photons/s/mrad^2^/mm^2^/0.1%. Other groups reported 210 nm wavelength^44^, broadband undulator radiation with a peak around 30 nm^45^, and 200 nm^46^ at similar bandwidths. We have recently been made aware of ref. ^47^, which provides a more detailed study of the undulator radiation presented in ref. ^46^. Here, the authors report on a plasma-driven undulator source with 7.6% FWHM bandwidth and a brightness of 6 × 10^17^ photons/s/mrad^2^/mm^2^/0.1%. The emitted wavelength is tuneable in the range of 200 nm to 300 nm by varying the undulator gap and electron beam energy.

Studying the generation of spontaneous, i.e., incoherent, undulator radiation using the plasma-generated electron beams available today, these experiments represent important steps towards the ultimate goal of a plasma-driven free-electron laser.

Previously reported undulator radiation often featured an on-target central wavelength, that was mainly determined by fluctuations of the electron beam energy. The resulting radiation bandwidths varied from shot-to-shot, due to instabilities in the electron beam properties, which limited the application of this radiation even the simplest experiments.

Here we show, plasma-driven undulator radiation tuneable in discrete steps over more than one octave from 13 nm to the water-window at 4 nm. Using a set of custom-designed multilayer mirrors, we effectively monochromatize the undulator radiation central wavelength and, as a result, provide discrete wavelengths at sub-nm precision with one-percent relative bandwidth. Our specific setup creates a photon pulse, which closely resembles the electron bunch length and charge profile, and thus opens a new path to study the laser-plasma acceleration mechanism.

## Results

### Experimental setup

**Figure 1:**
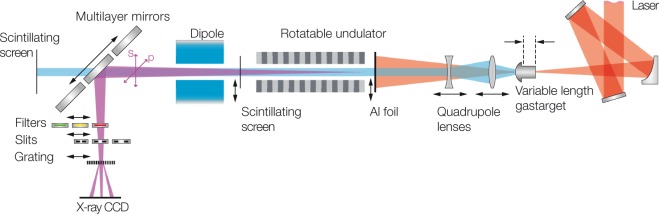
Experimental Setup. The drive laser (red) is focused into a variable-length hydrogen-filled plasma target generating electron beams in the range of 230 to 410 MeV. Residual laser light after the target is blocked by a 20 µm tick aluminium foil. The electron beam (blue) is captured and collimated by a fully motorized doublet of permanent magnet quadrupole lenses and then directed through a miniature undulator to generate x-ray pulses. A permanent magnet dipole disperses the electron beam onto a scintillating screen for spectral characterization and separates the x-ray (purple) and electron beam. X-ray pulses are effectively monochromatized and reflected by 90° off the beam axis into the x-ray spectrometer using a set of custom multilayer mirrors. The x-ray beam polarization is switched between s and p by rotating the undulator around the beam axis. A scintillating screen after the undulator can be moved into the beam to aid alignment and to characterize the transverse profile of the electron beam. With *z* being the axis along the electron and then x-ray beam path, the plasma target is located at *z* = 0 and the undulator entrance at *z* = *46.5* cm. The scintillating screen is located at *z* = *144.5* cm, the mirrors at *z* = *188.5* cm, and the x-ray CCD chip at *z* = *288.5* cm.

To generate wavelength-tuneable few-nm plasma-driven undulator radiation, we use the Atlas TW-class Ti:Sapphire laser system (see Methods) in an experimental setup illustrated in Fig. 1. The laser is focused into a variable-length hydrogen-filled plasma cell^40,48,49^, where it drives a plasma wave to generate electron beams in the self-injection regime. The electron beams are collimated using a pair of in-vacuum permanent-magnet quadrupole lenses^50^ and are then propagated through an undulator, i.e., a periodic arrangement of dipoles, which forces electrons onto a sinusoidal trajectory. In their mean rest frame, the oscillating electrons emit dipole radiation which is Doppler-shifted in the laboratory frame, where the observed on-axis radiation wavelength λ follows the well-known equation

1 $$\lambda =\frac{{\lambda }_{u}}{2{\gamma }^{2}}(1+\frac{{K}^{2}}{2}).$$

Here, *K* is the dimensionless undulator parameter, which characterizes the strength of the on-axis magnetic field, *λ*_*u*_ is the magnetic field period, and $\gamma =E/m{c}^{2}$ is the normalized electron beam energy. As *λ* scales quadratically with *γ*, changing the electron beam energy enables tuning of the undulator radiation over a broad spectral range. Undulator radiation is linearly polarized. In our setup, the compact undulator can be rotated around the beam axis to provide x-rays of s- and p-polarization.

After the undulator, a permanent magnet dipole disperses the electron beam onto a scintillating screen to measure the electron beam spectrum and to separate the x-ray from the electron beam. Following the dipole magnet, curved multilayer mirrors can selectively be moved in the x-ray beam path to focus the undulator radiation into a spectrometer based on a gold transmission grating^51^. The undulator radiation bandwidth, *Δλ*, scales with the electron beam spectral width, i.e., $\Delta \lambda /\lambda  \sim 2\Delta \gamma /\gamma$, and is initially very broad due to the large electron energy spread, *Δγ*. The multilayer mirrors effectively monochromatize the undulator radiation at a well-defined wavelength and any instabilities inherited from the electron beam are converted into a single parameter: the photon number per pulse. We use three custom multilayer designs, which are optimized for a narrowband reflectivities around 13.0 nm, 6.2 nm, and 4.2 nm, respectively. Each multilayer mirror is combined with a thin metal filter of appropriate transmission window to suppress parasitic visible and scattered laser light, as well as possible dipole radiation from the electron spectrometer dipole.

### Few-nm undulator radiation

To generate undulator radiation of a particular wavelength, our experimental procedure is as follows. A key factor enabling tuning of the X-ray wavelength is control over the electron energy. Thus, we first optimize the plasma density and target length, i.e., the field gradient inside the plasma cavity and the total acceleration distance, to generate the desired electron energy according to Eq. (1). Limited by the available drive laser parameters, the plasma target supports an electron peak energy tuneable in the range of 230 to 410 MeV. Second, we adopt the beam transport and account for the energy-dependent focal length of the fixed-gradient quadrupole lenses by adjusting their longitudinal position to collimate the selected target electron energy through the undulator. Using a multilayer mirror to transport the generated undulator radiation further monochromatizes the emitted spectrum and effectively provides a fixed wavelength and bandwidth at the detector. Following this procedure, we stepwise tune the emitted wavelength from 13 nm, to 6 nm, to 4 nm in a single experiment shift.

**Figure 2:**
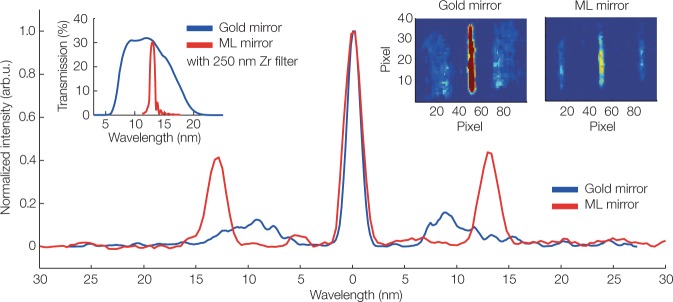
Effect of multilayer mirrors on the undulator spectrum. Single-shot undulator radiation reflected from a multilayer mirror centred at 13 nm (red), and broadband radiation using a gold-coated mirror (blue) covering 7 to 14 nm, are both measured with a 250 nm Zr filter and a 500 µm entrance slit. The spectra are lineouts from the raw CCD camera data (insets top right). The multilayer mirror effectively monochromatizes the initially broadband undulator radiation. The relative bandwidth is 3% rms after the mirror, verified by independent characterization of the multilayer mirror. In this lineout, the resolution is limited by the spectrometer entrance slit. The inset (top left) shows the effective transmission from undulator to target, i.e., the measured mirror reflectivity combined with the transmission of a 250 nm Zr filter. Note, that both spectra are normalized to the 0^th^ diffraction order of each spectrum, respectively. Since it reflects a broader bandwidth than the multilayer mirror, the intensity of the 0^th^ diffraction order in the gold mirror setup is higher and thus causes a lower normalized signal level of the 1^st^ diffraction order.

Using an electron energy of 230 MeV, we expect to generate undulator radiation at 13 nm, or 95 eV. The corresponding measured single-shot undulator radiation spectra are shown in Fig. 2, using a gold-coated mirror (blue) and a multilayer mirror (red) to transport the photons from the undulator to the spectrometer.

The gold-coated mirror reflects over a wide spectral range and the detected undulator radiation is broadband, Δλ/λ ≈ 0.3. The bandwidth is determined by the chromaticity of the electron beam optic, which effectively acts as a bandpass filter (ΔE/E ≈ 0.15) for the electron beam^43^, see also Supplementary Fig. 3.

In contrast, the multilayer mirror acts as a monochromator and thus provides a reproducible central x-ray wavelength and bandwidth independent of the stability of electron beam parameters. The spectrum measured with the multilayer mirror has a ±1^st^ diffraction order at 13.0 nm and a shot-to-shot reproducibility of 0.2 nm rms, which verifies this effect. The relative bandwidth of 3% rms is set by the multilayer mirror reflectivity, which has been independently characterized by the soft x-ray reflectometer at the Advanced Light Source (ALS)^52^. An experimental characterization of all custom multilayer mirrors is presented in Supplementary Fig. 1.

Note, that the width of the ±1^st^ diffraction orders, shown in Fig. 2, is dominated by the 500 µm size of the spectrometer entrance slit and therefore appears to be broader than 3%.

**Figure 3:**
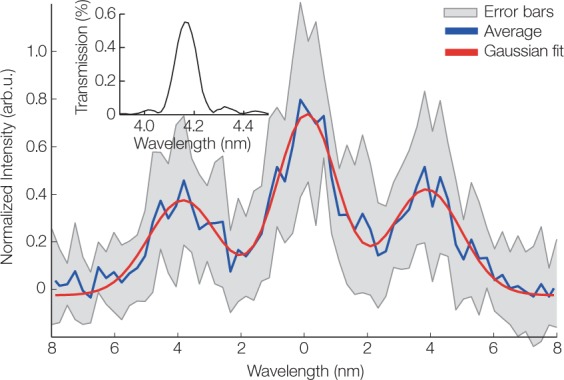
Water-Window Spectrum. Undulator radiation of 4.2 nm wavelength. The presented spectrum (blue) is averaged over 10 shots (standard deviation in grey), due to the low efficiency of the photon transport from undulator to detector. A fit of three independent Gaussians (red) to the 0^th^ and ±1^st^ diffraction order shows a wavelength of 3.9 nm, a deviation of less than 10% of the multilayer design wavelength, which is well within the alignment tolerance of the multilayer mirror featuring an angle-sensitive reflectivity. The relative bandwidth is 1% rms, as verified by an independent mirror characterization. In the presented spectrum, the bandwidth appears to be broader due to the limited, source-size dominated spectrometer resolution. The inset shows the transmission of the multilayer mirror in combination with a 200 nm Pd filter, causing, in combination with the 8% grating efficiency, a low single-shot signal at the detector.

In principle, the mirror reflectivity can be designed for any central wavelength and bandwidth in the energy regime specified above. For example, generating the corresponding electron energy, tuning the setup and using a tailored multilayer mirror, we measure undulator radiation at 6.2 nm (199.5 eV) and 1.6% relative bandwidth. Generating even shorter wavelengths, we reach into the water-window at 4.2 nm (296.6 eV), which is the highest photon energy from a plasma-driven undulator reported to date and shown in Fig. 3.

Here, the presented spectrum (blue) is averaged over 10 shots, as our specific multilayer design in combination with the metal filter and the spectrometer grating had a combined efficiency of only 0.04% causing a low signal on the detector. An improved mirror design^53^ would increase the signal level by a factor of 3 and using a gracing incidence geometry would further increase the photon transport efficiency (see Supplementary Fig. 2) in future measurements. The measured undulator radiation relative bandwidth at 4 nm is 1% rms, verified by an independent characterization of the multilayer mirror. X-rays are detected in 92% of the drive laser shots.

At 4 nm the number of emitted photons per pulse within 1% rms bandwidth is 2.5 × 10^5^ with a shot-to-shot fluctuation of 30% rms (see Methods), which agrees well with the shot-to-shot fluctuations in charge.

To provide an order-of-magnitude estimation for the peak brightness^54^ of our setup at 4 nm we use a previous characterization of the variable-length plasma target^48^, which reported a normalized emittance of 0.4 mm.mrad. Note, that as the undulator radiation at a certain wavelength is generated from electrons with a particular energy, the energy-resolved emittance is the quantity relevant for this discussion. With the beam optics set to collimate a 410 MeV beam, we calculate a beam size in the undulator of about 100 µm by 375 µm rms using a particle tracking code. Assuming a 5-fs bunch length, which was independently measured for the variable-length plasma target^40^, and with 10^4^ photons in 0.1% bandwidth emitted from the undulator, we estimate an upper limit for the peak brightness of 10^19^ photons/s/mrad^2^/mm^2^/0.1% bandwidth^54^. The on-target brightness is lower due to losses in the photon transport. Due to the energy spread, it is difficult to experimentally verify the electron beam size, which is a dominating factor in the calculation of the undulator brightness. Electrons off the design energy are not matched to the beam transport and feature a large beam size. In the experiment, the beam size is measured after the undulator using a scintillating screen. A scintillating screen cannot resolve different energies and all electrons contribute to the measured spot, which is therefore larger compared to a monochromatic 410 MeV beam relevant for emission at 4 nm. An over-estimated electron beam size results in an under-estimated photon brightness. Using only the electron beam size measured at the scintillating screen, we conservatively derive a peak brightness of order 10^17^ photons/s/mrad^2^/mm^2^/0.1% bandwidth at 4 nm from the undulator. This range for the estimated brightness is consistent with results reported by other groups for a laser-plasma undulator source^42–44^. Due to the variations in photon flux, we expect a shot-to-shot fluctuation of the beam brightness by at least 30% rms, which, however, we could not directly verify experimentally due to the above-mentioned constraints.

## Discussion

Laser-driven x-ray sources, including high harmonic generation^18,19^, laser-driven undulators^42–46^, betatron radiation^26–28^ and Compton scattering^32,33^, each are based on fundamentally different physical mechanisms, typically cover distinct and complementary ranges of the x-ray spectrum, and provide different performance in key characteristics, such as the peak and average photon flux, coherence, the pulse repetition rate, and the scalability towards shorter wavelengths. It is therefore difficult, and beyond the scope of this work, to directly compare laser-based techniques. Eventually, laser-based sources will form a set of complementary tools, each providing specific benefits for certain applications. Here we discuss the prospects and potential of a laser-plasma driven undulator.

Using laser-plasma accelerated, GeV-level electron beams available in experiments today, plasma-driven undulator radiation can, in principle, cover a range from few to sub-nm wavelengths, while providing a high peak photon flux at a Hz-level repetition rate. The performance is mainly determined by the available drive laser repetition rate and the electron beam quality. The photon bandwidth scales with the beam energy spread providing 10%-level bandwidths for currently available laser-plasma beams. Any quality improvement in the electron beam directly benefits the generated photon bandwidth.

In our experiment, the photon energy of the undulator radiation was limited by the maximum electron beam energy we could achieve, which was, in turn, limited by the available energy of the laser pulses. To further reduce the undulator radiation bandwidth to a 1% level, we applied spectral filtering using custom multilayer mirrors.

Increasing electron energies to the GeV-level, which has already been demonstrated in several laboratories^34–37^, would enable us to extend our source into the few Angstrøm regime. For example, we expect of order 10^4^ photons on target at 7 Å (1.8 keV), 1% rms bandwidth and few-fs pulse length using a 1 GeV electron beam with our current undulator and an already fabricated and characterized multilayer mirror of 50% reflectivity. While our current setup is limited to a stepwise tuning owing to the discrete wavelengths supported by the multilayer mirrors, a future extension with a monochromator based on a multilayer mirror pair could provide continuous wavelength tuning.

Plasma-driven undulator radiation offers, in principle, new possibilities to diagnose the temporal properties of the electron beam, which is difficult to access using other techniques^55^. The undulator pulse duration is a good representation of the electron bunch duration if the slippage between the electrons and the generated x-rays in the undulator is limited. This condition requires a short undulator, i.e., a small number of undulator periods *N*_*u*_, and a wavelength *λ* much shorter than the electron bunch length *σ*_*z*_, i.e., a few-nm wavelength for an electron bunch of few-fs duration such that ${N}_{u}\times \lambda \ll {\sigma }_{z}$.

With a mirror of broadband reflectivity, e.g., a gold-coated mirror, the full spectrum from a plasma-driven undulator pulse can be transported to an interaction point, to measure the photon pulse length as a representative of the electron bunch length. Laser-based streaking, for example, is a well-established technique in attosecond pulse metrology^19^ and has previously been used to characterize x-ray pulse durations^56,57^.

In principle, one could also gain access to longitudinal electron phase space. We propose to repeatedly measure the undulator pulse length using multilayer mirrors of increasing bandwidth. A chirped electron bunch would result in a measured undulator pulse length that is proportional to the bandwidth of the selected multilayer mirror. To perform such a measurement, we would require an additional set of custom design multilayer mirrors, each having a slightly different bandwidth. Note, that our setup already fulfils the important requirements ${N}_{u}\times \lambda \ll {\sigma }_{z}$, i.e., providing low slippage between the electron bunch and the undulator pulse.

In its current configuration, our setup generates synchrotron-like, i.e., incoherent, undulator radiation. Compared to state-of-the-art high-harmonic sources, the repetition rate of our setup is low and limited by the drive laser.

Ultimately, the goal of the community is to operate a plasma-driven undulator in the free-electron laser regime^29–31^, providing a compact source of high-brightness coherent few-fs x-rays. Free-electron laser operation, however, requires exceptional electron beam quality, which is not yet available from a laser-plasma accelerator. Studying the spontaneous emission regime, demonstrating key features of plasma-driven undulator radiation, offering new paths for advanced electron beam diagnostics, and thereby advancing the field are important steps in the development of a laser-plasma driven free-electron laser.

## Methods

### Electron beams

We use the Atlas Ti:Sapphire laser system (1.5 J in 28 fs FWHM, corresponding to a peak power of ~50 TW) at the Max-Planck-Institute for Quantum Optics (Garching, Germany), focused with a f/20 off-axis parabola to a spot size of 20 µm and providing an intensity of 6.4 × 10^18^ W/cm^2^, corresponding to a normalized intensity of a_0_ = 1.7. The laser operates with a repetition rate of one shot every few seconds, determined by the gas load from the plasma target. Electrons beams are generated in a self-injection regime using a variable-length (3–7 mm) plasma cell target, described and characterized in refs. ^48,49^. Filled with hydrogen up to a pressure of 130 mbar, corresponding to a plasma density of 3.2 × 10^18^ cm^−3^, and operated with optimized parameters^48^ the target provides electron beams with up to ~2 mrad FWHM divergence. Due to geometrical constraints the spectrometer can only measure electron energies above 200 MeV. Above 200 MeV, the electron beam spectrum contains 50 pC of charge. The electron spectra are broadband and feature a high-energy peak. During the experiment, the electron peak energy, *E*, is tuned in the range from 230 to 410 MeV by varying the target gas pressure and length. The electron beam optic is chromatic and acts as a bandpass filter, *ΔE/E* ≈ 0.15 for the electron beam^43^.

We model the electron beam optics using the Elegant^58^ and Madx codes and tune the longitudinal position of the quadrupoles for each electron beam energy to collimate the beam through the undulator. The permanent magnet quadrupole lenses have focal lengths of f_1_ = 11.4 cm and f_2_ = −17.3 cm for a 410 MeV beam. To measure the electron spectra a permanent magnet dipole of 40 cm length and on-axis field of 0.9 T disperses the electron beam on a charge-calibrated^59^ scintillating screen.

*X-Rays*: We use a 60-period miniature undulator (undulator period λ_u_ = 5 mm, undulator parameter K = 0.37) to generate undulator radiation. As the undulator has a fixed gap, the wavelength has to be tuned by changing the electron beam energy. The undulator radiation is collected either using an in-house gold-coated mirror, or a set of in-house manufactured multilayer mirrors. The multilayer mirrors are produced with a dual ion beam sputtering system at the Max-Planck-Institute of Quantum Optics, generating a sequence of molybdenum/silicon or chromium/scandium layers^60,61^ on spherical super-polished fused silica substrates with 2 m radius of curvature. We achieve a surface roughness and interface roughness on the order of 0.4 nm, which is crucial to obtain high reflectivities. The layer design and the 45° angle of incidence tailors the reflectivity of the mirror to a specific central wavelength and bandwidth according to the Bragg equation and the number of contributing layer pairs. The mirror reflectivities have been independently measured at the reflectometry beamline at the Advanced Light Source (Berkeley, USA). We characterize the undulator radiation with a transmission grating based spectrometer. A filter wheel equipped with thin metal filters of aluminium (Al, 300 nm thick), zirconium (Zr, 250 nm thick), palladium (Pd, 200 nm thick), and indium (In, 250 nm thick), reduces residual stray light from the electron drive laser and pre-selects the wavelength range of the spectrometer. The spectrometer offers slit sizes of 100 µm, 250 µm and 500 µm width. By reducing the slit size, we trade spectral resolution for photon flux on the detector. The x-rays are dispersed by 1008 l/mm free-standing gold transmission grating^51^ onto the x-ray CCD camera, which has a 1^st^ diffraction order efficiency of 8% in the wavelength range of interest. We use a Princeton Instruments Pixis XO-2048B and a Pixis XO-400B CCD camera, of 13.5 × 13.5 µm^2^ and 20 × 20 µm^2^ (XO-400B) pixel size and cool the chip below −20 °C to reduce thermal noise. To confirm that the generated signal is undulator radiation, we record a spectrum using a gold-coated broadband mirror and verify that the ±1^st^ diffraction order shows the characteristic and well-known red-shift with observation angle, i.e., $\lambda \propto {\theta }^{2}$. The photon number per pulse is determined summing the photon counts on the CCD image (with background subtracted) and accounting for the quantum efficiency of the chip, the transmission of the x-ray filters and the reflectivity of the multilayer mirrors. For these measurements, the entrance slit and the grating are removed from the x-ray beam path. At 4 nm, we obtain 2.5 × 10^5^ photons in 1% bandwidth, generated from 4 pC of charge in the contributing range of the electron spectrum. Using a simple analytical estimation for a thin (filament) electron beam resonantly emitting on axis^54^ this charge agrees with the measured number of photons within a factor of 2. We attribute this discrepancy to uncertainties in the spectrometer calibration and the emission from the thick electron beam. Gamma ray hits (hot pixels) are eliminated from the raw CCD image using a threshold filter. For the analysis presented in Fig. 2, we additionally apply a 3 × 3 pixels median filter to the camera images (shown as insets). The lineouts are taken after the hot pixel removal. To measure x-ray spectra at 4 nm wavelength we remove the entrance slit and bin 4 × 4 camera pixels for increased sensitivity and reduced noise level.

## Supplementary information

Supplementary Information.

## Data Availability

The data is available from the corresponding authors upon reasonable request.
