# Supplementary material for: Water-Window X-Ray Pulses from a Laser-Plasma Driven Undulator
Source: Sci Rep. 2020 Mar 27;10:5634. doi: 10.1038/s41598-020-62401-4 (PMC7101387; doi:10.1038/s41598-020-62401-4)
Supplement: Supplementary file 1 — Supplementary Information. [file 41598_2020_62401_MOESM1_ESM.pdf]

Water-Window X-Ray Pulses from a Laser-Plasma Driven Undulator

Supplementary Material

A. R. Maier[*,1,2,3], N. Kajumba[1,2], A. Guggenmos[1,2], C. Werle[1], J. Wenz[1,2], N. Delbos[1], B. Zeitler[1], I. Dornmair[1], J. Schmidt[1,2], E. M. Gullikson[4], F. Krausz[1,2], U. Schramm[5], U. Kleineberg[1,2], S. Karsch[1,2], and F. Grüner[1,2,3]

[1] *Ludwig-Maximilians-Universität, Department Physik,*
Am Coulombwall 1, 85748 Garching, Germany

[2] *Max-Planck-Institut für Quantenoptik,*
Hans-Kopfermann-Str. 1, 85748 Garching, Germany

[3] *Center for Free-Electron Laser Science and Department of Physics Universität Hamburg,*
Luruper Chaussee 149, 22761 Hamburg, Germany

[4] *Center for X-Ray Optics, Lawrence Berkeley National Lab,*
1 Cyclotron Road, Berkeley, CA 94720, USA

[5] *Helmholtz-Zentrum Dresden - Rossendorf, Institute of Radiation Physics,*
Bautzner Landstrasse 400, 01328 Dresden, Germany

* corresponding author: andreas.maier@cfel.de

Mirror Reflectivities

The custom-design multilayer mirrors used in the experiment have been independently characterized at the reflectometry beamline at the Advanced Light Source. Supplementary Figure S1 shows the measured reflectivity (blue solid) for mirrors with a central energy of 95 eV (13.0 nm), 199.5 eV (6.2 nm) and 295.6 eV (4.2 nm). The reflected bandwidth is 3 eV for every mirror, corresponding to relative bandwidths of 3.0 % , 1.6 % and 1.0 %, respectively. The mirrors effectively monochromatise the incident undulator radiation, providing a well defined wavelength and bandwidth. However, multilayer mirrors typically have a large refelectivity in the visible. To suppress residual laser light, stray light, and ambient light, we combine the multilayer mirrors with thin metal film filters. These filters block visible wavelengths but feature a distinct broadband x-ray transmission. The red solid lines in Supplementary Figure S1 show the x-ray transport efficiency, provided by the mirror in combination with a 250 nm zirconium (Zr) filter (a), and a 200 nm paladium (Pd) filter (b and c).

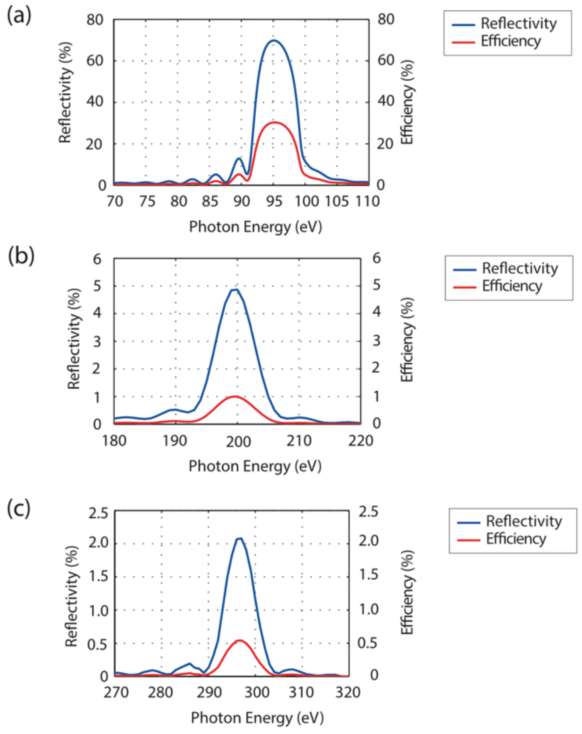

Supplementary Figure S1 | Reflectivities. Measured multilayer mirror reflectivity (blue solid) and the x-ray transport efficiency provided by the mirror in combination with a 250 nm Zr filter (panel a), and 200 nm Pd filter (panel b and c).

Improved Mirror Design

A different mirror design [50], which was unfortunately not available during our experiment, provides a reflectivity of 10 % at 300 eV, which represents a factor of 3 improvement in reflectivity.

We can further boost the mirror reflectivity to 27 % at 300 eV using a new layer design, shown in Supplementary Figure 2, and operating at an angle of incidence of 70°. This new multilayer mirror has a bandwidth of 4.7 eV rms, and, in combination with a 200 nm Pd filter, provides a transport efficiency (undulator to target) of 7 %, which is over an order of magnitude improvement. Future experiments will greatly benefit from this configuration.

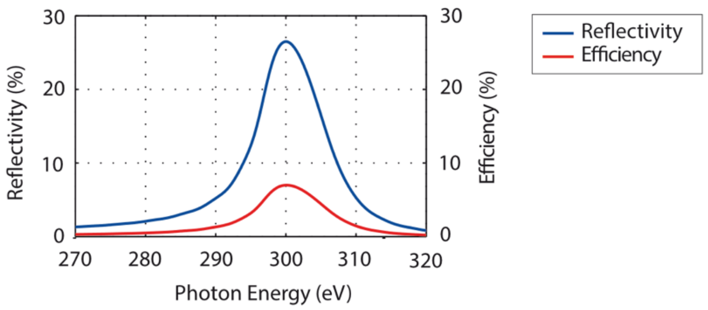

Supplementary Figure S2 | Improved mirror design. Using a new layer design at 70° angle of incidence, we boost the mirror reflectivity to about 27 % at 300 eV. In combination with a 200-nm Pd filter, the transport efficiency from undulator to target is 7 %.

Typical electron spectrum

The electron beam optic isolates a peak around the set-point energy [43] from the broadband background. For our measurements, the electron beam focus was set onto the x-ray CCD camera, which disturbes the electron spectrum measured with a scintillating screen after the permanent magnet dipole.

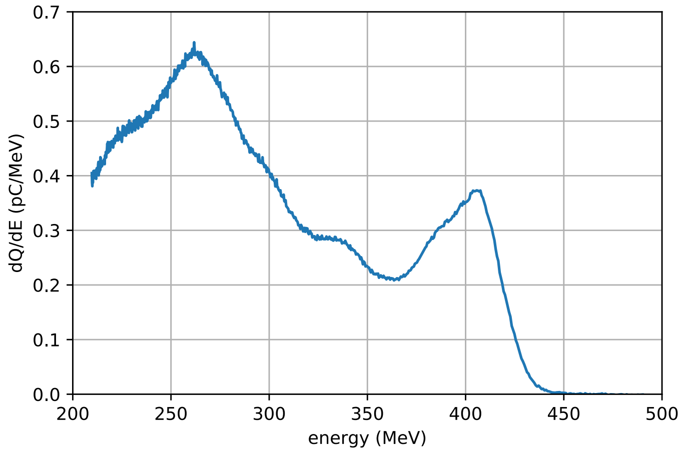

Supplementary Figure S3 | Typical electron spectrum from a 4 nm x-ray run. The beam optic effectively acts as a filter function for the electron beam [43] isolating a peak around the set-point energy from the broadband background.
